# Exercise Improves Clock Drawing Test in Older Adults with Kidney Disease

**DOI:** 10.1093/geroni/igaf122.2263

**Published:** 2025-12-31

**Authors:** Diego Redondo-Sáenz, Ulf Bronas

**Affiliations:** Columbia University School of Nursing, New York, New York, United States; Columbia University, New York, New York, United States

## Abstract

Older patients with chronic kidney disease (CKD) are at high risk of cognitive impairment. We have previously demonstrated a coexistence between higher levels of fitness and psychomotor speed and executive control in older adult patients with CKD. This study investigated the impact of a 6-month home-based walking intervention Vs usual care on the clock drawing test (CDT). The CDT evaluates select cognitive domains, including executive function. Participants (n = 39, mean age of 67.6 (5.0) years; 66.7% women, 71.8% Black) were randomized to intervention or usual care. The intervention included a wearable activity monitor, weekly didactic phone meetings, interactive tools (e.g., reminders), and monthly coach-delivered feedback. The compliance rate was 78%, the mean minutes of exercise per week was 159.9 (149.2) minutes/week. The CDT was scored using Libon’s criteria and ANCOVA was used for analysis adjusting for age and education. CDT total score and executive function error score trended higher in the intervention group, compared to the control group at 6-months with a mean difference of 0.48 (95% CI of -0.02-0.97, p=.06), and a mean difference of -.012 (95% CI -.122 to .098. p=.08) respectively. The effect sizes were large at η²=.11 and η²=.079 respectively. Total CDT score at 6-months was correlated with 6-minute walking distance (r=.36, p=.04) and executive function error score at 6-months was correlated with change in step count (r=-39, p=.03). These findings suggest that in older adults with CKD, a 6-months of home-based exercise walking may be associated with an improvement in executive function and constructional praxis.

